# Supplementary material for: Optimized detection of homologous recombination deficiency improves the prediction of clinical outcomes in cancer
Source: NPJ Precis Oncol. 2022 Dec 29;6:96. doi: 10.1038/s41698-022-00339-8 (PMC9800569; doi:10.1038/s41698-022-00339-8)
Supplement: Supplementary file 1 — REPORTING SUMMARY [file 41698_2022_339_MOESM1_ESM.pdf]

nature portfolio

Corresponding author(s): Anniina Farkkila

Last updated by author(s): Nov 23, 2022

Reporting Summary

Nature Portfolio wishes to improve the reproducibility of the work that we publish. This form provides structure for consistency and transparency in reporting. For further information on Nature Portfolio policies, see our Editorial Policies and the Editorial Policy Checklist.

Statistics

For all statistical analyses, confirm that the following items are present in the figure legend, table legend, main text, or Methods section.

n/a	Confirmed	
☐	☒	The exact sample size (*n*) for each experimental group/condition, given as a discrete number and unit of measurement
☐	☒	A statement on whether measurements were taken from distinct samples or whether the same sample was measured repeatedly
☐	☒	The statistical test(s) used AND whether they are one- or two-sided<br>*Only common tests should be described solely by name; describe more complex techniques in the Methods section.*
☐	☒	A description of all covariates tested
☐	☒	A description of any assumptions or corrections, such as tests of normality and adjustment for multiple comparisons
☐	☒	A full description of the statistical parameters including central tendency (e.g. means) or other basic estimates (e.g. regression coefficient) AND variation (e.g. standard deviation) or associated estimates of uncertainty (e.g. confidence intervals)
☐	☒	For null hypothesis testing, the test statistic (e.g. *F*, *t*, *r*) with confidence intervals, effect sizes, degrees of freedom and *P* value noted<br>*Give P values as exact values whenever suitable.*
☒	☐	For Bayesian analysis, information on the choice of priors and Markov chain Monte Carlo settings
☐	☒	For hierarchical and complex designs, identification of the appropriate level for tests and full reporting of outcomes
☐	☒	Estimates of effect sizes (e.g. Cohen's *d*, Pearson's *r*), indicating how they were calculated

Our web collection on statistics for biologists contains articles on many of the points above.

Software and code

Policy information about availability of computer code

Data collection	For pan-cancer samples, allele-specific copy number segments were obtained from the Genomics Data Commons (GDC) portal (https://portal.gdc.cancer.gov/). For OVA-TCGA analysis, allele-specific copy number segments, DNA methylation, gene-level copy number profiles (including gene deletions), and clinical information data were obtained from the GDC data portal. Complementary clinical information was obtained from the PanCanAtlas-GDC data portal. For PCAWG: allele-specific copy number segments, mutational drivers, and clinical information were obtained from the International Cancer Genome Consortium data portal (https://dcc.icgc.org/pcawg).
Data analysis	The statistics analysis was performed in R version 4.0.3

For manuscripts utilizing custom algorithms or software that are central to the research but not yet described in published literature, software must be made available to editors and reviewers. We strongly encourage code deposition in a community repository (e.g. GitHub). See the Nature Portfolio guidelines for submitting code & software for further information.

Data

Policy information about availability of data

All manuscripts must include a data availability statement. This statement should provide the following information, where applicable:

- Accession codes, unique identifiers, or web links for publicly available datasets
- A description of any restrictions on data availability
- For clinical datasets or third party data, please ensure that the statement adheres to our policy

Sequencing data for the DECIDER and TERVA cohort is available through the European Genome-Phenome Archive under the study accession number: EGAS00001006775

Human research participants

Policy information about studies involving human research participants and Sex and Gender in Research.

Reporting on sex and gender	Due to the disease, all patients were female.
Population characteristics	Age; residual tumor after surgery was categorized as present or absent, Progression-free survival and overall survival were reported from public sources or collected from medical records (DECIDER, TERVA). For DECIDER cohort the anatomical site and timing of sample retrieval was collected from medical records.
Recruitment	The patients for DECIDER and TERVA were recruited according to the ethical regulations approved by the local IRB, and all patients signed an informed, voluntary consent.
Ethics oversight	For the DECIDER and TERVA cohorts, the Ethics Committee of the Hospital District of Southwest Finland approved both studies (Dnro: 145 /1801/2015).

Note that full information on the approval of the study protocol must also be provided in the manuscript.

Field-specific reporting

Please select the one below that is the best fit for your research. If you are not sure, read the appropriate sections before making your selection.

☒ Life sciences ☐ Behavioural & social sciences ☐ Ecological, evolutionary & environmental sciences

For a reference copy of the document with all sections, see nature.com/documents/nr-reporting-summary-flat.pdf

Life sciences study design

All studies must disclose on these points even when the disclosure is negative.

Sample size	No sample size estimations were performed in this article. The data used correspond to retrospective and prospective cohorts.
Data exclusions	For the pre-annotation of HRD/HRP samples in OVA-TCGA the HRP sample TCGA-13-1511 was annotated as "undefined" as an outlier in the number of total AIs. For OVA-TCGA, only patients disease treated with cisplatin and/or carboplatin were selected. For PCAWG, data from all patients were used (no treatment information available). For the optimization of TNBC in TCGA, the samples 0d7cde44-ec86-415a-8667-0ea894d1c344 and f1b4f790-083e-44f0-b924-06dc7c167a20 were annotated as "undefined" as outliers in the number of total AIs.
Replication	For ovaHRDsca we used OVA-TCGA samples as training set and the PCAWG, DECIDER and TERVA as validation cohorts. For tnbcHRDscar, we used TCGA's TNBC samples as reported by Lehmann et al, 2016; and the cohort reported by Staaf et al 2019 as validation.
Randomization	The randomization was not relevant for study.
Blinding	Not relevant for this study.

Reporting for specific materials, systems and methods

We require information from authors about some types of materials, experimental systems and methods used in many studies. Here, indicate whether each material, system or method listed is relevant to your study. If you are not sure if a list item applies to your research, read the appropriate section before selecting a response.

Materials & experimental systems

n/a	Involved in the study	
☒	☐	Antibodies
☒	☐	Eukaryotic cell lines
☒	☐	Palaeontology and archaeology
☒	☐	Animals and other organisms
☐	☒	Clinical data
☒	☐	Dual use research of concern

Methods

n/a	Involved in the study	
☒	☐	ChIP-seq
☒	☐	Flow cytometry
☒	☐	MRI-based neuroimaging

Clinical data

Policy information about clinical studies

All manuscripts should comply with the ICMJE guidelines for publication of clinical research and a completed CONSORT checklist must be included with all submissions.

Clinical trial registration	Not relevant for the study.
Study protocol	Not relevant for the study, as it was not a clinical trial.
Data collection	For the DECIDER cohort details can be found at: https://www.deciderproject.eu/. For the TERVA cohort details can be found at: https://www.healthcampusturku.fi/terva-project/ . The rest of the cohorts correspond to publicly available data.
Outcomes	See section Human research participants.
