# Supplementary material for: Optimized detection of homologous recombination deficiency improves the prediction of clinical outcomes in cancer
Source: NPJ Precis Oncol. 2022 Dec 29;6:96. doi: 10.1038/s41698-022-00339-8 (PMC9800569; doi:10.1038/s41698-022-00339-8)
Supplement: Supplementary file 2 — Supplementary Material [file 41698_2022_339_MOESM2_ESM.pdf]

Supplementary Figure 1

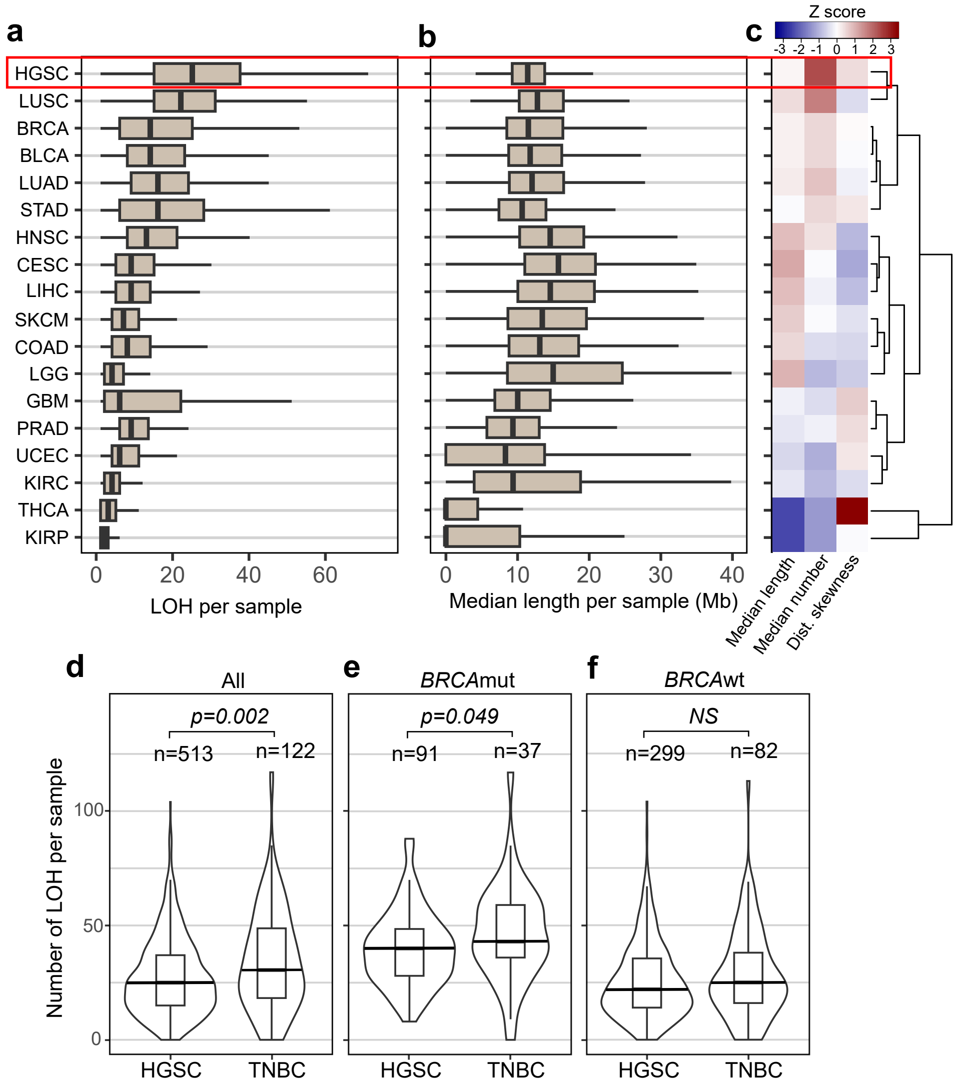

Supplementary Figure 1. Pan-cancer characterization of LOH shows unique patterns in HGSC. **a** Box plots showing the number of LOH events larger than 3Mb and smaller than 50Mb in the different cancer types. **b** Box plots showing the median length of LOH events (longer than 3Mb and smaller than 50Mb) in the cancer types. The black vertical lines represent the sample medians, the boxes extend from first to third quartile and whiskers indicate the values at 1.5 times the interquartile range, no outliers are shown. **c** Hierarchical clustering of the cancer types using the median length, median number of LOH events per sample, and the skewness of the distribution of LOH length. **d** Violin- and box plots representing the number of LOH events in all HGSC samples as compared to TNBC (U test, p=0.005). Long horizontal lines represent the medians further notations are similar as in panel a .**e** Violin and box plots representing the number of LOH events in *BRCA*mut HGSC samples as compared to TNBC (U test, p=0.021). **f** Violin and box plots representing the number of LOH events in *BRCA*wt HGSC samples as compared to the TNBC (U test, NS).

Supplementary Figure 2

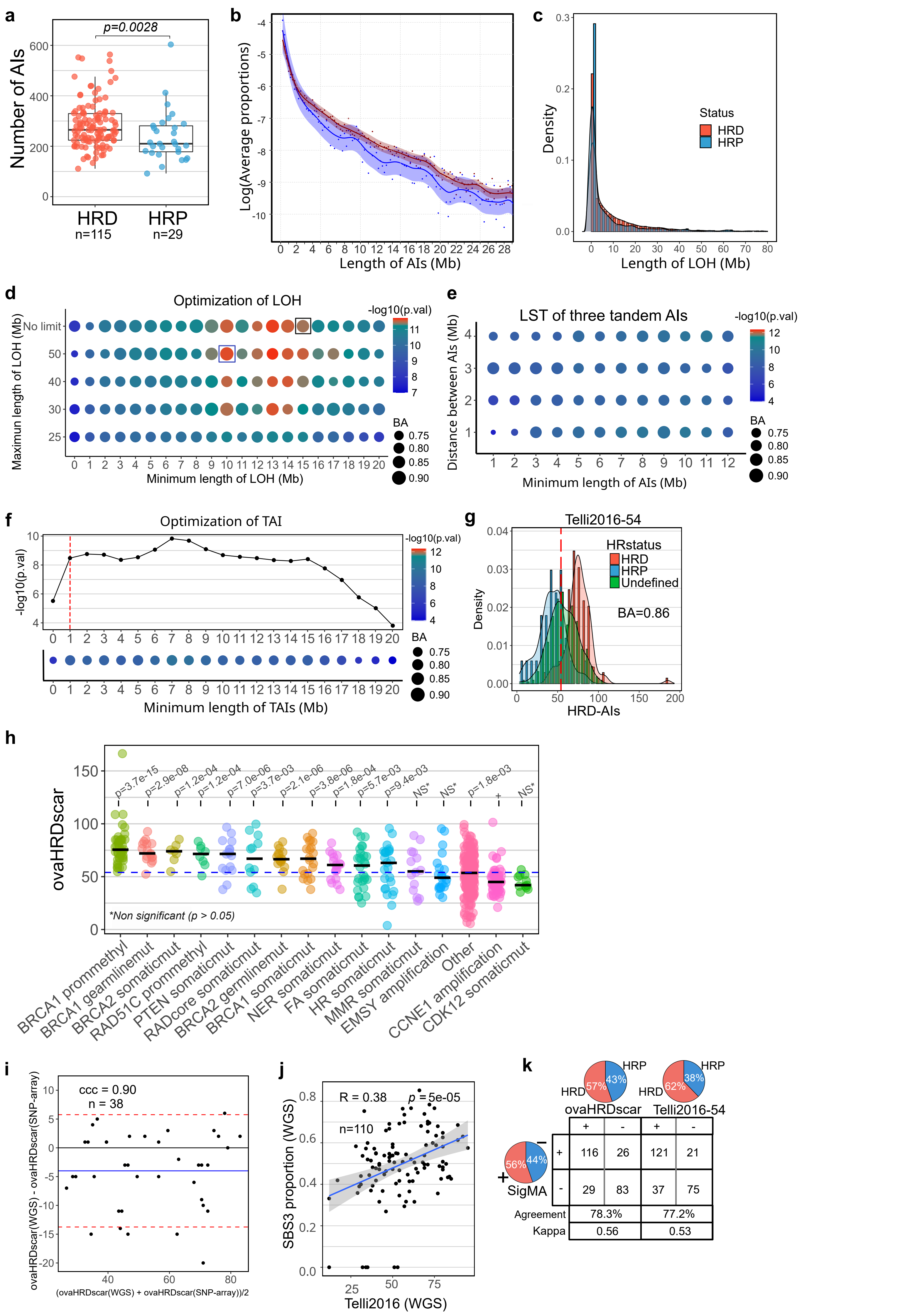

Supplementary Figure 2. Descriptive statistics of HRD and HRP in HGSC. a Box plot showing the number of allelic imbalances in HRD (red) and HRP (blue) samples in OVA-TCGA. The black horizontal lines represent the sample medians, the boxes extend from first to third quartile and whiskers indicate the values at 1.5 times the interquartile range. **b** The average proportion of segments (AIs) equal or greater than the given length in HRD (red) and HRP (blue) samples, the blue and red lines correspond to smoothing using cubic splines, confidence intervals are shown in shaded colors. **c** Density distribution of LOH events in HRD (red) and HRP (blue) samples. **d** The accuracy of the new LOH criteria (blue boxes) and those utilized in Telli et al. (black box); the size of dots represents the decision tree balanced accuracy (BA) when using the corresponding cut-off, colors correspond to the statistical difference in abundance of AIs between HRD versus HRP samples (U test p value). **e** Accuracy of HR classification using three tandem allelic imbalances LSTs of a given minimum length (x axis) and distance between them smaller than 1 to 4 Mb (y axis). Dot sizes and colors are presented similarly as in panel d. **f** Upper panel: Visualization of the statistical difference (U test p-values) in the abundance of TAIs between HRD versus HRP samples for selected TAIs length. Lower panel: The dot sizes and colors in the lower panel correspond to the description in panel d and e. **g** Density distribution of HRD-AIs according to Telli2016 algorithm, the red dashed line represents a cut-off vale of 54 to define the HR-status. The balanced accuracy (BA) of classifying the annotated HRD and HRP is shown, density distribution colors correspond to the samples annotated as in Fig. 2a. **h** OVA-TCGA samples stratified by genomic alterations and their corresponding ovaHRDscar levels. U test p values are shown for the comparison of ovaHRDscar levels between the corresponding alterations as compared to the samples with CCNE1 amplification. Horizontal lines represent the median values per group. **i** Bland-Altman plot that shows the concordance (Concordance correlation coefficient, CCC = 0.90) between the number of ovaHRDscars detected using SNP-arrays and WGS in the intersecting samples from OVA-TCGA and PCAWG. Blue line represent the average difference and the red dotted lines the 95% confidence intervals of the mean. **j** Correlation (Pearson, r=0.38) between the SBS3 proportion in WGS data from PCAWG versus the number of scars using the Telli2016 approach. Blue line shows the regression line, and the 95% confidence intervals are shown in grey. **k** The SBS3 status inferred using SigMA16 showing a higher agreement with ovaHRDscar (agreement=78.3%, Cohen's kappa = 0.56) than with the Telli2016 algorithm using an HRP/HRP cut-off value of 54 (agreement=77.2%, Cohen's kappa = 0.53). In the pie charts and table + and - correspond to the number of HRD positive and HRD negative samples identified under each criterion, respectively. On the bottom is shown the number of samples and the level of agreement between the corresponding criteria.

Supplementary Figure 3

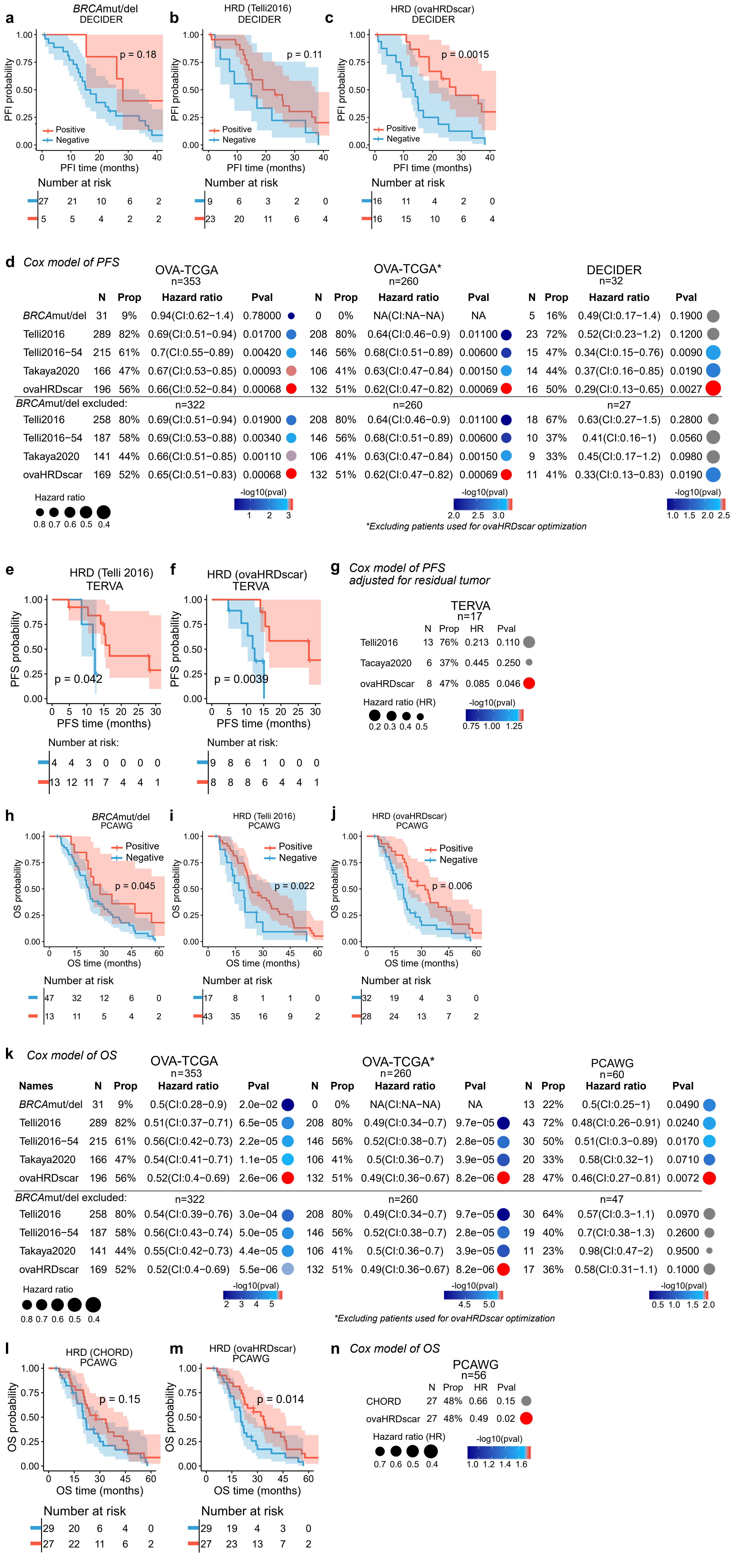

Supplementary Figure 3. ovaHRDscar shows an improved prediction of PFS and OS in HGSC patients. a to c Kaplan-Meier plots for PFS in the DECIDER cohort. The patients were stratified using different criteria: the *BRCAmut*/del status in the left panel (**a**), the Telli2016 algorithm in the middle panel (**b**) and the ovaHRDscar algorithm (**c**). **d** Cox regression models for PFS in HGSC patients using different selection criteria. The colors, rows and columns descriptions are the same as in Figure 3d. **e to f** Kaplan-Meier plots of PFS in the prospective TERVA cohort. The patients were stratified using: The Telli2016 algorithm on the left (**e**), the ovaHRDscar on the right (**f**). **g** Cox regression models for PFS in the TERVA cohort. The patients were stratified using different criteria. The colors, rows and columns descriptions are the same as in Figure 3d. **h to j** Kaplan-Meier plots for OS in the DECIDER cohort stratified using the different criteria: *BRCA*mut/del status on the left (**h**), the Telli2016 algorithm (**i**) and the ovaHRDscar algorithm (**j**). **k** Cox regression models for OS adjusted for patient age at diagnosis in HGSC patients stratified using different criteria similarly as in Figure 3i. **l to m** Kaplan-Meier plots for OS in the PCAWG cohort stratified using: the CHORD signature on the left (**l**), the ovaHRDscar on the right (**m**). **n** Cox regression models for OS in the PCAWG cohort. Patients were stratified using CHORD and ovaHRDscar criteria; the dot colors, rows and columns descriptions are the same as in Figure 3d.

Supplementary Figure 4

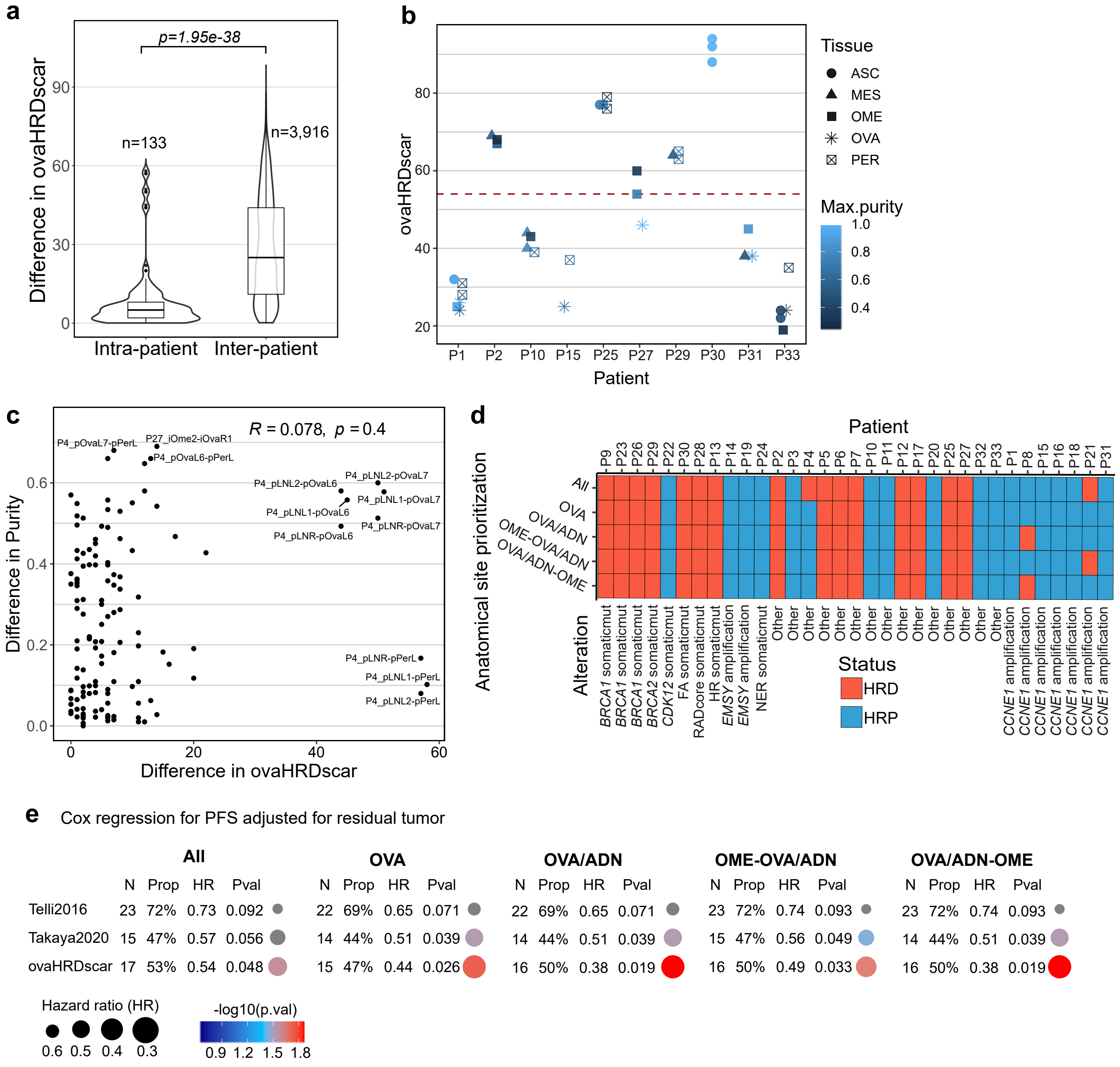

Supplementary Figure 4. Inter and intra-patient variability of ovaHRDscar levels. a Left box plot for the difference of ovaHRDscar values between all possible intra-patients' samples pairs. Right box plot for the corresponding difference of ovaHRDscar values in all possible inter-patients' samples pairs in the DECIDER prospective cohort. The black horizontal lines represent the sample medians, the boxes extend from first to third quartile and whiskers indicate the values at 1.5 times the interquartile range. U-test p-value is shown. **b** Samples from different anatomical sites with tumor purity and ovaHRDscar levels indicated. **c** The correlation between the difference of ovaHRDscar values between all intra-patient sample pairs and the difference in tumor purity of the corresponding sample pairs. Patient P4 was ignored as an outlier. **d** Color table of HR-status classification and HR-related genomic alterations using five different approaches to prioritize anatomical sites for ovaHRDscar calculations. **e** Cox regression models for PFS adjusted for the residual tumor after surgery in the DECIDER cohort using different algorithms and five different anatomical site prioritizations. Colors, rows and column descriptions are the same as in Figure 3d.

Supplementary Figure 5

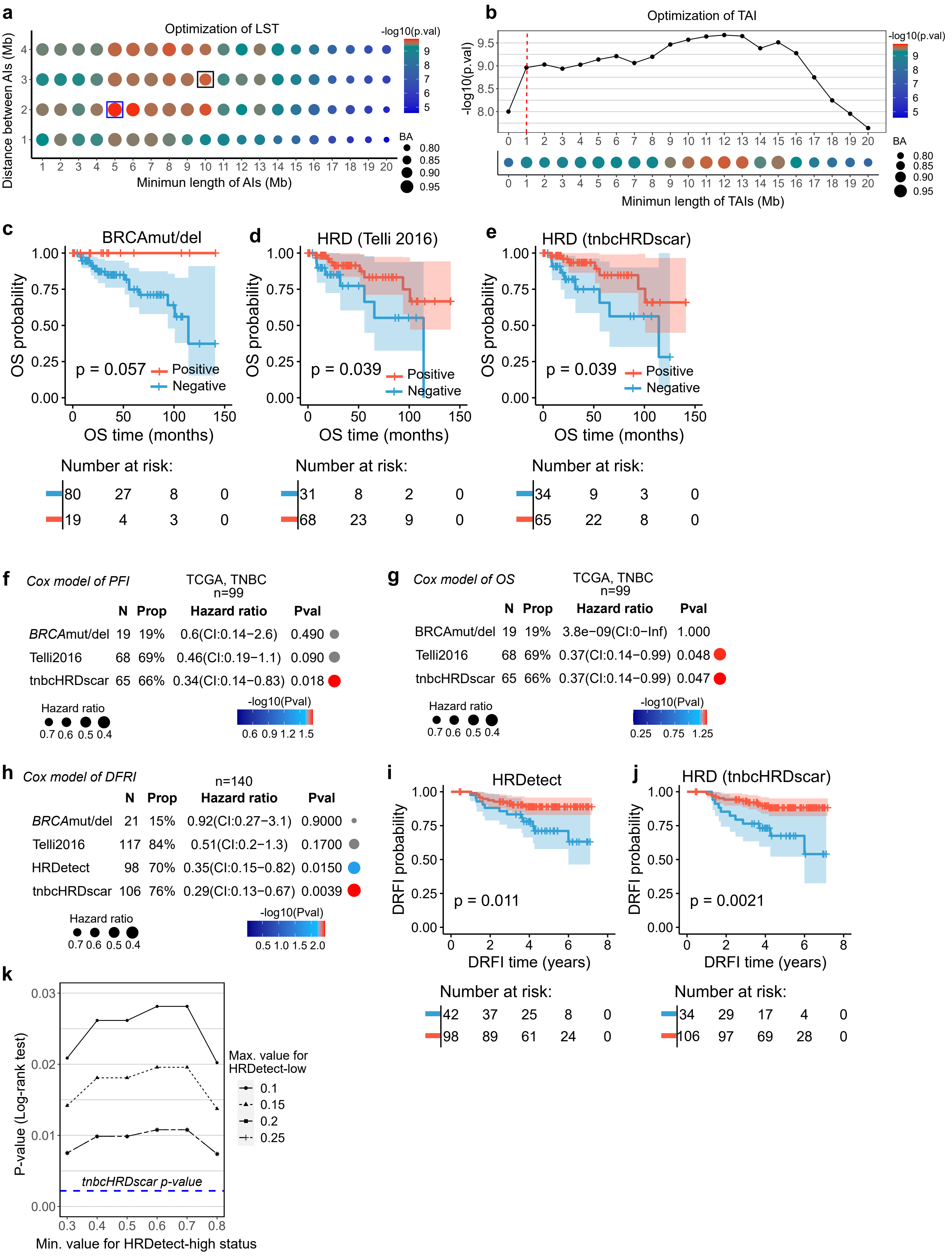

Supplementary Figure 5. Machine learning-aided detection of HRD-AI in TNBC improves the prediction of clinical outcomes. a Generation of HRD-LST events. The size of the dots represents the decision tree balanced accuracy (BA) of the classification of HRD and HRP when selected LSTs with the corresponding criteria. The dot colors correspond to the statistical difference in abundance of the selected LSTs between HRD versus HRP samples (U test, p-value). The black box corresponds to the selection criteria proposed by Telli2016, blue box corresponds to the tnbcHRDscar BA and U test value. **b** Upper panel: Visualization of the change in p-values (U test) when selecting TAIs >1Mb (red dashed line). Lower panel: the difference in abundance of TAIs of selected length between HRD versus HRP samples. The dot sizes and colors in the lower panel correspond to the description in panel a. **c to e** Kaplan-Meier plots for OS in the TCGA's TNBC patients stratified using the different criteria; **c** BRCAmut/del status on the left, **d** the Telli2016 algorithm, and **e** the tnbcHRDscar algorithm. **f** Cox regression models for PFS in HGSC patients stratified using different criteria, the dot colors descriptions are the same as in Figure 3d. **g** Cox regression models for OS in in the TCGA's TNBC patients stratified using the different criteria. **h** Cox regression models for DRFI in an independent TNBC patient cohort[21] stratified using different criteria. **i to j** Kaplan-Meier plots for DRFI in an independent TNBC patient cohort[21] stratified using: HRDetect based on whole genome sequencing data (**i**), the tnbcHRDscar based on SNP-array data (**j**). **k** Selection of different values to define the HRDetect-high/low status for patient stratification in the TNBC patient cohort[21] and its association with DRFI (Log-rank test, p-value). Patients with intermediate HRDetect values were ignored. In blue line, the Log-rank p-value when using tnbcHRDscar in panel j.
